# How do intestinal probiotics restore the intestinal barrier?

**DOI:** 10.3389/fmicb.2022.929346

**Published:** 2022-07-14

**Authors:** Hong-Zhong Gou, Yu-Lin Zhang, Long-Fei Ren, Zhen-Jiao Li, Lei Zhang

**Affiliations:** ^1^The First Clinical Medical College, Lanzhou University, Lanzhou, China; ^2^Department of General Surgery, The First Hospital of Lanzhou University, Lanzhou, China; ^3^Key Laboratory of Biotherapy and Regenerative Medicine of Gansu Province, The First Hospital of Lanzhou University, Lanzhou, China

**Keywords:** intestinal barrier, probiotics, tight junctions, mucins, restore mechanism

## Abstract

The intestinal barrier is a structure that prevents harmful substances, such as bacteria and endotoxins, from penetrating the intestinal wall and entering human tissues, organs, and microcirculation. It can separate colonizing microbes from systemic tissues and prevent the invasion of pathogenic bacteria. Pathological conditions such as shock, trauma, stress, and inflammation damage the intestinal barrier to varying degrees, aggravating the primary disease. Intestinal probiotics are a type of active microorganisms beneficial to the health of the host and an essential element of human health. Reportedly, intestinal probiotics can affect the renewal of intestinal epithelial cells, and also make cell connections closer, increase the production of tight junction proteins and mucins, promote the development of the immune system, regulate the release of intestinal antimicrobial peptides, compete with pathogenic bacteria for nutrients and living space, and interact with the host and intestinal commensal flora to restore the intestinal barrier. In this review, we provide a comprehensive overview of how intestinal probiotics restore the intestinal barrier to provide new ideas for treating intestinal injury-related diseases.

## Introduction

The intestinal barrier is an important line of defense to maintain the homeostasis of the intestinal microenvironment and is divided into mechanical, chemical, immune, and microbial barriers, which interact to maintain intestinal homeostasis (Camilleri et al., 2012). Intestinal microbes are an essential part of the intestinal barrier and help maintain normal intestinal barrier function. These microbes are abundant in the host intestine, which they colonize in a symbiotic manner. Some intestinal microbes act as probiotics, that is, they are beneficial to the host’s health. The Food and Agriculture Organization of the United Nations and the WHO define probiotics as “live microorganisms, which when administered in adequate amounts confer a health benefit on the host” (Hill et al., 2014; Araya et al., 2015). Binda et al. (2020) proposed the minimum standards required to correctly use the term “probiotics.” Probiotics must have “strain characteristics, intended use safety, clinical trials, and the ability to survive at effective doses within the product’s shelf life.” They have numerous functions, including participating in food digestion, promoting intestinal motility, synthesizing vitamins, decomposing harmful substances, and enhancing the intestinal barrier (Vitetta et al., 2018; Rose et al., 2021; Tang et al., 2021). With the development of molecular biology, genetic engineering, fermentation culture, microfluidics, and experimental models in recent years, it has been possible to use single or compound probiotics to restore the intestinal barrier, providing a new clinical treatment option for intestinal barrier damage-related diseases.

## Intestinal mechanical barrier

The intestinal mechanical barrier comprises various intestinal epithelial cells and intercellular junction complexes differentiated from intestinal stem cells (ISCs) localized at the bottom of crypts (Capaldo et al., 2017; Ge et al., 2020). Probiotics can increase the expression of genes and proteins involved in tight junction (TJ) signaling, regulate the apoptosis of intestinal epithelial cells (IECs), and induce the proliferation of IECs to restore the intestinal mechanical barrier (Ohland and Macnaughton, 2010; Sharma et al., 2010; Ashida et al., 2011; La Fata et al., 2018).

### Probiotics affect the TJs between IECs

The TJs between IECs can selectively transport substances, prevent pathogenic bacteria and harmful substances from entering the intestinal lumen, and maintain normal intestinal barrier function (Paradis et al., 2021). The TJ-related complex proteins of IECs are intracellular and membrane proteins. Some of the intracellular proteins are zonula occludens (ZO)1, ZO2, ZO3, and cingulin (Balda and Matter, 2000; Suzuki, 2020). Membrane proteins include a variety of transmembrane proteins in TJs, such as the occlusive protein (occludin), synaptic connexin (claudin), and junction adhesion molecules (JAMS; Colegio et al., 2003; Campbell et al., 2017). Probiotics affect the intestinal barrier function by regulating the expression of genes and proteins involved in TJ signaling in IECs (Table 1).

**Table 1:** List of probiotics that affect the tight junction to restore the intestinal mechanical barrier.

Probiotic	Experimental subject	Gene/protein expression increased (↑) or decreased (↓)	References
*Escherichia coli* Nissle1917	Germ-free mice	ZO1↑ gene and protein expression	Ukena et al., 2007
*Lactobacillus reuteri* (LR1 R2LC, 4,659, I5007 DSM 17938)	IPEC-1 cells DSS colitis mice OVA-sensitized rat	ZO1↑ ZO1 redistribution Occludin ↑claudin1, 3, 5, 7, 8, 9↑ gene and protein expression	Cario et al., 2004; Yang et al., 2015; Ahl et al., 2016; Wang et al., 2016; Yi et al., 2018; Tulyeu et al., 2019
*Lactobacillus rhamnosus* GG	T84 cell line mice	ZO1↑Occludin↑ protein expression ZO1 redistribution Claudin1 redistribution	Johnson-Henry et al., 2008; Zhang et al., 2020
*Lactobacillus plantarum* MB452	Caco-2 cells	Occludin↑ ZO1↑ Claudin2↑ gene and protein expression	Anderson et al., 2010
*L. plantarum* WCFS1	Healthy volunteers Caco-2 cells	Occludin↑ ZO1↑ gene and protein expression	Karczewski et al., 2010
*Bifidobacterium infantis* ATCC No. 15697 *Lactobacillus acidophilus* ATCC No. 53103	Caco-2 cells	Occludin ↑Claudin1↑ protein expression	Guo et al., 2017
*B. infantis* strain BB-02	NEC mice	Claudin4 and occludin redistribution	Bergmann et al., 2013
*L. plantarum* CGMCC No.1258	Caco-2 cells	Occludin↑ ZO1 ↑ Claudin1↑ JAM1↑ protein expression	Qin et al., 2009
*Bacillus subtilis* CW14	Caco-2 cells	ZO1↑ Claudin1↑ protein expression	Peng et al., 2019
*L. acidophilus* LA1	Caco-2 cells DSS colitis mice	Occludin↑ TLR2↑ protein expression	Al-Sadi et al., 2021
Probiotic mixture (*Bifidobacterium infants*, *L. acidophilus*, *Enterococcus*, *Bacillus cereus*)	NEC mice	Occludin↑ Claudin1↑ protein expression	Zhao et al., 2021
*S. boulardii* CNCM I-745	T84 cell line	ZO1 distribution	Pontier-Bres et al., 2015

Ukena et al. (2007) found that when *Escherichia coli* Nissle1917 (EcN 1917) colonized the intestinal tract of germ-free mice, the gene and protein expression of the TJ molecule ZO1 increased, thereby increasing the TJ structure between IECs and decreasing intestinal permeability, which could improve intestinal epithelial barrier function. ZO1 expression also increased after transplanting EcN 1917 into colitis mice. However, this does not suggest that EcN 1917 has the same effect in the human intestinal environment, and the establishment and maintenance of the intestinal defense system may be a requirement for bacterial colonization and adhesion.

*Lactobacillus reuteri* maintains the integrity of the intestinal barrier by increasing the expression of TJ proteins in IECs. Wang et al. (2016) revealed that *L. reuteri* LR1 could mitigate enterotoxigenic *E. coli* (ETEC)-induced membrane barrier damage by maintaining the correct localization of ZO1 and inhibiting the destruction of ZO1 protein. However, Yi et al. (2018) discovered that *L. reuteri* LR1 may increase the gene and protein expression of the TJ molecules ZO1 and occludin through the myosin light chain kinase signaling pathway, thereby alleviating the damage of intestinal epithelial barrier integrity caused by ETEC K88 infection. Ahl et al. (2016) found that *L. reuteri* can also increase the expression of the TJ proteins, occludin and ZO1, strengthen the intestinal barrier, and ameliorate dextran sulfate sodium (DSS)-induced colitis. Tulyeu et al. (2019) revealed that *L. reuteri* could prevent the decrease of TJ proteins expression in IECs caused by ovalbumin sensitization and substantially increase the protein expression of ZO-1; occludin; and claudin1, 3, 5, 7, 8, 9, and 15 in rat IECs, thereby effectively ameliorating the intestinal mucosal barrier function.

*Lactobacillus rhamnosus* affects TJ protein expression to restore the intestinal mechanical barrier. Johnson-Henry et al. (2008) found that *E. coli* O157:H7 caused abnormal distribution of ZO1 and claudin1 in polarized epithelial cells, as well as a decrease in ZO1 expression, resulting in increased permeability and decreased barrier function in an *in vitro* cell model. They cultured *L. rhamnosus* GG with polarized epithelial cells to induce the redistribution of ZO1 and claudin1 and increased expression of ZO1, improving barrier function. The postbiotic HM0539 from *L. rhamnosus* GG can enhance the resistance of mice to *E. coli* O157:H7 infection by attenuating the destruction of TJ proteins (Zhang et al., 2020).

*Lactobacillus plantarum* MB452 increases the gene and protein expression of ZO1, ZO2, occludin, and cingulin and regulates the expression of TJ protein-degrading genes (such as itchy E3 ubiquitin protein ligase and snail family transcriptional regressor 1), which stabilizes TJs and improves intestinal barrier function (Anderson et al., 2010). Furthermore, the reduction in proteasome gene expression induced by *L. plantarum* MB452 may be an additional mechanism to enhance TJ integrity. Karczewski et al. (2010) found that *L. plantarum* WCFS1 increases the expression of occluding- and ZO1-coding genes. They injected *L. plantarum* WCFS1 strain into the duodenum of healthy subjects and found that TLR2 pathway activation affected the expression and distribution of TJ proteins. Another study revealed that *L. plantarum* regulates protein levels and distribution of claudin1, occludin, JAM1, and ZO1 in an *in vitro* model, protecting Caco-2 cells from enteroinvasive *E. coli* and improving intestinal barrier function (Qin et al., 2009).

*Bifidobacterium infantis* and *Lactobacillus acidophilus* normalize the expression of the TJ proteins, occludin and claudin1, in an *in vitro* Caco-2 intestinal epithelial cell model, preventing barrier damage due to IL-1 stimulation (Guo et al., 2017). Furthermore, In a neonatal mouse necrotizing enterocolitis (NEC) model, *B. infantis* modulates the proper localization of claudin 4 and occludin in TJs, attenuates intestinal permeability, protects intestinal barrier function, and reduces the incidence of NEC (Bergmann et al., 2013). Peng et al. (2019) revealed that *Bacillus subtilis* CW14 could mitigate the damage of intestinal epithelial cell microvilli and the TJ proteins, ZO1 and claudin1, caused by ochratoxin A, and maintain genome stability. *L. acidophilus* LA1 considerably enhances the intestinal TJ barrier mediated by a TLR2 heterodimeric complex. LA1 increases the expression of the TJ protein occludin, but also prevents DSS-induced downregulation of occludin expression in mouse intestinal tissues and enhances TJ structure (Al-Sadi et al., 2021).

In addition to a single strain that can restore the intestinal barrier and improve the intestinal barrier function, a mixture of probiotics can also restore the intestinal barrier. Zhao et al. (2021) confirmed that the probiotic mixture (*B. infantis*, *L. acidophilus*, *Enterococcus*, and *Bacillus cereus*) could enhance the expression of claudin-1 and occludin, by regulating the pregnane X receptor-c-Jun N-terminal kinase signaling pathway, and ameliorate intestinal barrier damage in neonatal necrotizing enterocolitis. Furthermore, co-culture of *Saccharomyces boulardii* CNCM I-745 with T84 cells *in vitro* revealed that *S. boulardii* altered the distribution of ZO1 and maintained the barrier function (Pontier-Bres et al., 2015).

### Probiotics affect IEC apoptosis and proliferation

Intestinal probiotics can regulate the apoptosis of IECs, promote the proliferation of IECs, mitigate intestinal damage, and restore the intestinal mechanical barrier (Odenwald and Turner, 2017; Alam and Neish, 2018). *L. rhamnosus* GG promotes the proliferation of IECs by secreting the protein p40. p40 upregulates disintegrin and metalloproteinase domain-containing protein 17 catalytic activity to phosphorylate epidermal growth factor receptor and activate the phosphatidylinositol 3-kinase (PI3K)/protein kinase B signaling pathway, reduces apoptosis, and preserves barrier function (Yan et al., 2013; Shen et al., 2018; Capurso, 2019). Hou et al. (2018) demonstrated that *L. reuteri* D8 stimulates lamina propria lymphocytes to secrete IL-22 *via* aryl hydrocarbon receptor, and then activates the phosphorylation of signal transducer and activator of transcription 3 (STAT3) to promote the proliferation of IECs and increase the growth of intestinal organs, thus recovering the intestinal mucosal barrier, which restores the structural damage to the intestinal epithelium caused by TNF treatment. In a mouse model of inflammatory bowel disease (IBD), continuous administration of *B. subtilis* during the remission period can maintain the integrity of the intestinal barrier by regulating the proliferation of IECs and alleviate IBD (Liu et al., 2021). *Lactobacillus casei* and *Clostridium butyricum* can also promote the proliferation of IECs and restore the intestinal barrier (Ichikawa et al., 1999).

## Intestinal chemical barrier

The intestinal chemical barrier comprises mucin (MUC), antimicrobial peptides (AMPs), digestive fluids, lysozymes, mucopolysaccharides, other chemicals, and antimicrobial substances (Ren et al., 2019; Ge et al., 2020). Mucins are divided into transmembrane mucins and gel-forming mucins (Johansson and Hansson, 2016). The predominant membrane mucins in the intestine of humans and mice are MUC13 and MUC17 (Layunta et al., 2021), while MUC2 is the major secretory mucin in the gastrointestinal tract and is a major component of intestinal mucus (Tailford et al., 2015; Johansson and Hansson, 2016). MUC2 is the main component of the intestinal chemical barrier, covering IECs to form an intestinal mucus layer, which improves food absorption, provides attachment sites for intestinal symbiotic bacteria, and limits the combination of pathogens and IECs. AMPs are a type of polypeptide secreted by Paneth cells (Schoenborn et al., 2019) and have bactericidal, anti-inflammatory, immunity-improving, and tissue restoration-promoting effects (Bakshani et al., 2018). Several studies have shown that probiotics can regulate the expression of mucin, affect the formation of the mucus layer, and maintain the intestinal barrier function.

The increase in MUC2 expression by *L. acidophilus* A4 and its cell extracts significantly inhibited the attachment of *E. coli* O157:H7 to HT-29 IECs (Kim et al., 2008). Similar results were obtained when treating colorectal adenocarcinoma (Caco-2) cells with *L. acidophilus* LA1 (Bernet et al., 1994). In an *in vitro* model, *L. casei* GG increased the expression of MUC2 gene and protein to inhibit the translocation of specific pathogenic bacteria (Mattar et al., 2002). Caballero-Franco et al. (2007) revealed that the probiotic mixture VSL#3 (*Lactobacillus*, *Bifidobacterium*, and *Streptococcus*) increased the gene expression level of *MUC1*, *MUC2*, and *MUC3*.

Cazorla et al. (2018) found that the oral administration of *L. casei* CRL 43 and *L. paracasei* collection Nationale de cultures de microorganisms (CNCM) I-1518 to mice could increase the number of Paneth cells in the small intestine, and the AMPs released by Paneth cells could kill microorganisms, reduce the translocation of pathogens in the mucosa, and induce a barrier against intestinal infection. Ogawa et al. (2001) found that lactic acid bacteria and *L. casei* can also secrete acetic and lactic acids to reduce intestinal pH, inhibit the growth of pathogens, promote the balance of intestinal flora, and maintain the intestinal barrier function.

## Intestinal immune barrier

The intestinal immune barrier comprises gut-associated lymphoid tissue (GALT) and immune cells in the intestine. In the intestinal epithelial mucosa, propria contains Peyer’s node and immune cells from the innate and adaptive immune system, such as thymus (T) and bone marrow-or bursa-derived (B) cells, dendritic cells, and macrophages, together with the antibacterial peptides secreted by Paneth cells and secretory immunoglobulin A (IgA) from plasma cells. These participate in the immune defense mechanism of the intestinal barrier (Wells et al., 2017).

### Probiotics and immune cells

Probiotics can directly or indirectly regulate immune and anti-inflammatory functions. *In vivo* experiments in mice demonstrated that *Lactobacillus* can upregulate the expression of major histocompatibility complex II and the costimulatory cluster of differentiation (CD) 86, CD80, and CD40 and promote the maturation of dendritic cells (Drakes et al., 2004). *B. infantis* promotes the maturation of dendritic cells and accumulation of tolerogenic CD103 dendritic cells in GALT, which further regulates T-cell differentiation, induces anti-inflammatory factor expression, and improves intestinal mucosal immune response (Konieczna et al., 2013; Fu et al., 2017). EcN 1917 regulates T cell activation by affecting the T cell cycle and apoptosis to maintain intestinal immune homeostasis (Sturm et al., 2005). Jiang et al. (2019) found that *L. reuteri* induced macrophage activation in mice and enhanced macrophage phagocytosis to improve the immune function of the intestinal mucosa. *L. plantarum* 8,826 from the National Collection of Industrial Food and Marine Bacteria enhances the activity of T cell subsets and natural killer cells and increases the activity of the cytokine IL-10 *in vitro* (Dong et al., 2012). Kaushal and Kansal (2014) demonstrated that *L. acidophilus* and *B. bifidum* increased the phagocytic potential of aged mouse macrophages to improve immunity. A recent probiotic study showed that *L. rhamnosus* can reduce intestinal ischemia–reperfusion injury in mice by activating IL-10 release from macrophages through the TLR2 receptor signaling pathway (Hu et al., 2022).

### Probiotics regulate IgA secretion

Probiotics stimulate plasma cells to secrete IgA, but probiotic-induced IgA secretion may be strain-specific. Wang et al. (2019) reported that *C. butyricum* and *Enterococcus faecalis* increase the level of IgA in pig serum and improve immunity. Another study on probiotics found that after gavage of mice with *E. faecalis* CECT7121 increased intestinal mucosal IgA levels and enhanced local mucosal immune responses (Castro et al., 2016). El Hadad et al. (2019) showed that different concentrations of *B. bifidum* significantly stimulated the production of immune IgA in the intestinal mucosa of mice. Long-term consumption of fermented milk containing *L. casei* DN-114001 increased the amount of IgA in the large intestine and proved to be beneficial to the immune system of the intestinal mucosa (de Moreno de LeBlanc et al., 2008). *S. boulardii* was able to increase intestinal secretory IgA levels and modulate intestinal immunity in mice (Martins et al., 2009).

### Probiotic metabolites and intestinal immune factors

The metabolites of probiotics, such as short-chain fatty acids (SCFAs), can act on immune cells, such as mononuclear phagocytes and lymphocytes, affect the release of inflammatory factors and immune chemotaxis, and inhibit the proliferation of immune effector cells. Moreover, they can participate in immune regulation in the intestine (Kabat et al., 2014).

SCFAs can reduce the activity of the nuclear transcription factor NF-κB by inhibiting histone deacetylase; inhibit neutrophils and macrophages from releasing IL-8, tumor necrosis factor-α, and other inflammatory factors; and prevent chemotaxis of neutrophils to inflammation sites, thereby reducing intestinal inflammation (Tan et al., 2014). Kim et al. (2013) found that SCFAs activate mitogen kinase signaling through G protein-coupled receptors on intestinal epithelial cells, promote the production of immune factors, improve intestinal immune function in mice, and enhance intestinal barrier function.

## Intestinal microbial barrier

The intestinal microbial barrier is a bacterial membrane barrier formed by the commensal gut microbiota tightly adhering to the surface of the intestinal epithelial mucosa (Kayama et al., 2020). Intestinal probiotics are an important part of the intestinal microbial barrier, which helps regulate the balance of the number and structure of intestinal microflora. Probiotics can compete with pathogens for nutrients, and through space barriers, they competitively inhibit the attachment sites of targeted cells or the spread of microcolonies to resist the invasion of pathogens (Corr et al., 2009; Hynönen and Palva, 2013; Ge et al., 2020).

*L. rhamnosus* and *L. acidophilus* can competitively attach to the adhesion sites of HEp-2 cells and T84 cells, reducing the adhesion sites on the cell surface and preventing the invasion of pathogenic microorganisms, such as EPEC (Sherman et al., 2005). EcN can secrete a non-bacteriocin component to act on pathogenic microorganisms or host cells to weaken the adhesion of pathogenic microorganisms (Smajs et al., 2012). *Bacillus mesentericus*, *C. butyricum* and *E. faecalis* increase the diversity and abundance of intestinal microbes and maintain the balance of intestinal flora (Chen et al., 2010).

*L. plantarum* increased the abundance of *Bifidobacterium* and *Lactobacillus* in the cecum of mice treated with cyclophosphamide and decreased the abundance of *E. coli* and *Enterococcus* (Meng et al., 2019). Additionally, the mixture of *Lactobacillus fermentum* GOS57 and *L. plantarum* GOS42 was able to reduce the number of *Enterobacteriaceae*, increase the abundance of *Lactobacillus*, adjust the balance of intestinal flora, and enhance the intestinal barrier function (Linninge et al., 2019). *Lactobacillus rhamnosus* and *L. bulgaricus* can increase the abundance of beneficial bacteria in the intestines, inhibit the growth of harmful bacteria, and help restore the imbalance of intestinal flora caused by antibiotics (Li et al., 2020). However, Li et al. (2019) revealed that the intestinal flora of children with repeated respiratory infections is imbalanced, where the number of *Bifidobacterium* and *Lactobacillus* significantly decreases, and the number of *E. coli* increases. The administration of *Bifidobacterium* quadruple live bacterial tablets can effectively increase the number of *Bifidobacterium* and *Lactobacillus* in infected children, thereby maintaining the balance of intestinal flora and reducing the incidence of infection.

## Experimental models for probiotic research

Research on probiotics regarding the restoration of intestinal barrier function should be verified in human trials, which would lead to the development of treatments to improve the intestinal barrier, alleviate symptoms, and allow patients to recover quickly from disease. The safety of probiotics in humans needs to be prioritized, and although experiments in other animals and *in vitro* tests can further our understanding of the mechanisms of action, the human gut is rich in microbial species, the crosstalk between host and microbes is complex, and the physiology of non-human animals differs from that of humans. Therefore, animal and *in vitro* experiments are not sufficient for predicting the function of probiotic microorganisms in humans, and inferences from animal models and *in vitro* experiments cannot be extrapolated to humans (Hill et al., 2014; Abid and Koh, 2019; Suez et al., 2019). Common experimental models for probiotic studies and their advantages and limitations are listed in Table 2.

**Table 2:** Advantages and limitations of common experimental models for probiotic research.

Experimental model	*In vivo* or *in vitro*	Advantages	Limitations	References
Germ-free mice	*In vivo*	Used to study the mechanism of action of single strains or mixed microorganisms and the effect of microorganisms on the physiological state of the host to enable the colonization of foreign flora, to explore the causal relationship between flora and disease, and to verify the role of specific flora in disease.	Although it is an *in vivo* experiment, unlike the complex environment of the human body, part of the microbiota colonized in the mouse gut has not yet been found in the human gut.	Yi and Li, 2012; Al-Asmakh and Zadjali, 2015
DSS colitis mice	*In vivo*	DSS-induced colonic inflammation causes damage to intestinal epithelial cells and destruction of the mucosal layer, resulting in the entry of bacteria, other antigens, and pro-inflammatory substances into the mucosa or submucosa in the intestinal lumen, thus triggering an inflammatory response. This model can simulate the process of probiotics entering the body for repair after the damage of intestinal barrier function.	There are differences in intestinal flora between mice and humans, and the model cannot fully simulate the complex pathology in humans.	Oh et al., 2014; Kiesler et al., 2015; Hasannejad-Bibalan et al., 2020
Caco-2 cells	*In vitro*	The structure forms tight junctions and microvilli, which are similar to those of human small intestinal epithelial cells, and can be used to observe the effect of strains on TJ.	Lack of properties expected in epithelial cells; the interactions between probiotics and gut microbiota cannot be studied.	Sun et al., 2008; Huang et al., 2020
IPEC-1/IPEC-J2 cells	*In vitro*	The porcine digestive system is highly similar to human digestive system and can be used to study probiotic oxidative stress, transmembrane transport, and microbiota adhesion experiments.	Cells are easily influenced by the culture medium. Cannot mimic the complex interactions between probiotics and hosts.	Parthasarathy and Mansfield, 2009; Brosnahan and Brown, 2012; Kahlert et al., 2016
T84 cells	*In vitro*	Similar in structure to normal intestinal epithelial cells, forming tight junction structures; used to study epithelial barrier function	Cells are easily influenced by the culture medium. Cannot mimic the complex interactions between probiotics and hosts.	Krishnan et al., 2016; Ren et al., 2020
HT-29 cells	*In vitro*	The morphological and physiological properties are similar to those of normal human intestinal epithelial cells; can be used in probiotic adhesion experiments and as an *in vitro* model of epithelial cell differentiation.	Cell culture is influenced by the culture medium. Cannot simulate the interaction between flora in the human gut and human disease states.	Truant et al., 2003

Although these experimental models cannot be extrapolated to humans, they can be used to study the mechanism of action of probiotic strains. The results of such experiments do have therapeutic value for disease models related to intestinal damage. Probiotics from such experiments are safe to use without side effects before clinical trials if they meet the expected therapeutic goals and are administered under clinic conditions. Recent studies have reported probiotics that positively affect intestinal barrier function (Bron et al., 2017; Rose et al., 2021), but the specific mechanisms still need to be elucidated. In the future, these models can be used to study the specific mechanisms of action of probiotics and the effect of single or mixed strains on certain intestinal immune cells, identify more strains that can improve intestinal barrier function, and reveal the biological properties of the strains.

## Mechanism of action of probiotics

After entering the intestinal tract, the effect of probiotics on intestinal barrier function is intricate, and the specific mechanism of action may vary depending on the strain. Furthermore, the beneficial effect may be a result of a combination of actions, which may be related to the enzymes or metabolites produced by specific strains. Previous research on the mechanism of action of probiotics has been relatively superficial, with much of the research limited to *in vitro* or animal experiments. Regarding intestinal barrier function, increasing evidence indicates that probiotics improve intestinal barrier function through TLR-like receptors (especially TLR2) and that probiotics can enhance host intestinal immunity through TLR-like receptor-related cytokines and signaling pathways (Castillo et al., 2011; Ryu et al., 2016; Paveljšek et al., 2021). In addition, myeloid differentiation factor (MyD88); nuclear factor-kappa-B (NF-κB); mitogen-activated protein kinase (MAPK); protein kinase C(PKC); protein PI3K; and the STAT, p38, and ERK1/2 signaling pathways may be related to intestinal barrier function (Patel and Lin, 2010; Thomas and Versalovic, 2010; Yousefi et al., 2019), and the effect of probiotics may result from the coordination of multiple signaling pathways.

For example, *L. rhamnosus* can stimulate GALT to induce an immune response, produce immune factors, and resist invasion by pathogenic microorganisms. Furthermore, *L. rhamnosus* and its effective components (surface-layer protein and exopolysaccharides) pass through TOLL-like receptors to mediate the regulation of NF-κB, MAPK, and extracellular signal-regulated kinase (ERK) signaling pathways to regulate intestinal cytokines (Good et al., 2014; Gao et al., 2017; Geng et al., 2021). Yang et al. (2021) found that *L. plantarum* may inhibit the activation of the p38 and ERK1/2 MAPK signaling pathways mediated by TLR-4 to combat the excessive activation of the innate immune response caused by ETEC K88. *Lactobacillus delbrueckii* CIDCA 133 improves 5-fluorouracil chemotherapy-induced mucositis by regulating inflammatory pathways through the TLR2/4/Myd88/NF-κB signaling pathway (Barroso et al., 2022). *Bacillus* enhances intestinal immune function and is associated with the TLR2/4/Myd88/NF-κB signaling pathway (Du et al., 2018). However, the specific immune mechanism requires further research.

Peroxisome proliferator activated receptor-gamma (PPAR-γ), a nuclear hormone receptor that can regulate intestinal inflammation, is mainly expressed in the colon and may be another target of probiotics to regulate the intestinal barrier (Dubuquoy et al., 2006; Marion-Letellier et al., 2009). Eun et al. (2007) reported that the inhibition of intestinal inflammatory mediator expression by *L. casei* may be associated with PPAR-γ activation. Another study found that the protective effect of probiotics on epithelial barrier function is dependent on PPAR-γ activation (Ewaschuk et al., 2007). However, there is still a paucity of studies on the relationship between probiotics and PPAR-γ.

Probiotics improve intestinal barrier function by not only inhibiting host intestinal inflammatory response but also altering the thickness, nature, and secretion of intestinal mucus. The composition of intestinal microorganisms affects the nature of intestinal mucus, and a possible mechanism is the expression of glycosyltransferases, which varies according to the type and number of strains. The entry of probiotics into the organism changes the composition of intestinal microorganisms, which can change the nature and increase the secretion of mucus (Paone and Cani, 2020). Reportedly, *L. reuteri* improves the intestinal barrier by increasing mucus thickness in a mouse model of colitis (Ahl et al., 2016). In addition, probiotics increase the expression and localization of TJ proteins and mucin-related genes.

## Intestinal flora and host, flora interactions

Host intestinal epithelial cells form a structural interface that separates the lamina propria from the intestinal lumen. Therefore, the intestinal microorganisms in the lumen are in close contact with epithelial cells. The intestinal epithelium can distinguish between commensal and pathogenic microbiota through pattern recognition receptors that activate inflammation-related signaling pathways to resist pathogen invasion (O'Callaghan and Corr, 2019). The interaction between the microbiota in the intestinal lumen and intestinal epithelial cells and immune cells affects intestinal immunity. Microbiota interferes with bacterial adhesion, colonization, and invasion by affecting the expression of mucin genes in host goblet cells and stimulate the expression of antimicrobial peptides secreted by Paneth cells to affect intestinal immunity (Malago, 2015). Host and microbial interactions in the intestinal lumen maintain the homeostasis of the intestinal microenvironment.

Supplementation of probiotics may increase the number of certain microbes in the gut or metabolites of some specific strains that may improve intestinal barrier function by modulating intestinal immunity, TJ fraction, mucins, and changes in the intestinal microenvironment. These effects may be mediated by a crosstalk mechanism between probiotic and commensal bacteria. For example, Sugahara et al. (2015) supplemented human gut microbes in model mice with *B. longum* and found increased production of fecal pimelate, biotin, and butyrate, which may be caused by crosstalk between *B. longum* and the intestinal commensal microbiota.

Probiotics can compete with potentially pathogenic bacteria for nutrients and adhesion sites and inhibit their growth. As microbes establish a balanced microecosystem in the intestine, pathogenic bacteria must compete for binding sites and nutrients to survive. This competition between probiotics and pathogenic bacteria may reduce the possibility of colonization by pathogenic bacteria, reducing the chance of developing infectious diseases in the intestine (Bermudez-Brito et al., 2012; Stavropoulou and Bezirtzoglou, 2020). Furthermore, the metabolites of probiotics, such as SCFAs, can regulate intestinal pH, increase mucin gene expression (Burger-van Paassen et al., 2009), increase mucus production, and change the nature of mucus to prevent the adhesion of pathogenic bacteria. Fermentation products can also regulate intestinal immunity (Halloran and Underwood, 2019; Ratajczak et al., 2019), the specific mechanism of which may result from the combined action of the strains and their metabolites with intestinal immune cells or immune signaling pathways.

## Conclusion

**Figure 1:**
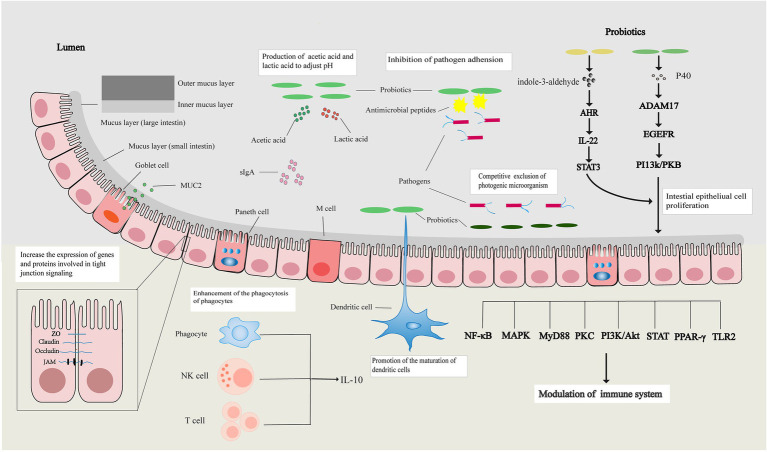
Probiotics regulate the intestinal barrier. Probiotics can increase the expression of tight junction-related genes and proteins, affect the apoptosis and proliferation of intestinal epithelial cells to restore the intestinal mechanical barrier, and restore the intestinal chemical barrier by increasing the expression of mucin and regulating intestinal pH. Probiotics also promote the maturation of immune cells, enhance the activity of immune cells, affect signal transduction pathways, and their metabolites promote the release of immune factors to restore the intestinal immune barrier. Probiotics restore the intestinal microbial barrier by regulating the balance of intestinal flora.

In summary, the intestinal barrier can separate substances from the intestinal cavity, prevent the invasion of pathogens, maintain the stability of the internal environment of the body, and protect the life and health of the human host. Probiotics can restore the intestinal mechanical, chemical, immune, and microbial barriers through various ways and maintain normal intestinal barrier function (Figure 1).

Presently, a myriad of studies has shown that probiotics can restore the intestinal barrier and treat intestinal injury-related diseases by enhancing TJs, increasing the expression of mucin, regulating the immune system, and inhibiting the adhesion of pathogenic bacteria. However, the mechanisms underlying the restoration of the intestinal barrier by probiotics have not been fully studied, and thus, more in-depth research is needed. Moreover, owing to the diversity of probiotics, limitations of the technology for extracting single probiotics, and complexity of the mechanism of compound probiotics, applying probiotics to the treatment of intestinal-related diseases by restoring the intestinal barrier faces ongoing challenges. With the development of biomarkers, genetic engineering, fermentation, separation, extraction technologies, and experimental design, researchers can further clarify the mechanism of probiotics at the genetic and molecular levels, make further breakthroughs in the screening, processing, and clinical application of probiotics, and develop more probiotics for the treatment of intestinal injury-related diseases.

## Author contributions

All authors made significant contributions to the conception and design of the study, participated in the drafting of the article and in the critical revision of the intellectual content, and approved the final manuscript. All authors contributed to the article and approved the submitted version.

## Funding

This study was supported by grants from the National Natural Science Foundation of China (31960236), Natural Science Foundation of Gansu Province (21JR7RA369), and the Talent Innovation and Entrepreneurship Project of Lanzhou City (2019-RC-34).

## Conflict of interest

The authors declare that the research was conducted in the absence of any commercial or financial relationships that could be construed as a potential conflict of interest.

## Publisher’s note

All claims expressed in this article are solely those of the authors and do not necessarily represent those of their affiliated organizations, or those of the publisher, the editors and the reviewers. Any product that may be evaluated in this article, or claim that may be made by its manufacturer, is not guaranteed or endorsed by the publisher.
